# Ecocomposite Filaments from Spent Coffee Grounds for FFF 3D Printing: Material Properties and Printability

**DOI:** 10.3390/polym18121453

**Published:** 2026-06-10

**Authors:** Jung-Tien Lo, Yu-Chen Chien, Teng-Chun Yang

**Affiliations:** Department of Forestry, National Chung Hsing University, Taichung 402, Taiwan; lrzhen9163542@gmail.com (J.-T.L.); g111033208@smail.nchu.edu.tw (Y.-C.C.)

**Keywords:** polylactic acid (PLA), spent coffee grounds (SCGs), 3D-printed biocomposites, fused filament fabrication (FFF), thermal properties, mechanical properties

## Abstract

In this study, spent coffee grounds (SCGs) were incorporated into polylactic acid (PLA) filaments and 3D-printed parts to investigate their effects on thermal, physical, and mechanical properties. Differential scanning calorimetry showed that SCG addition slightly reduced the glass transition temperature of PLA while markedly increasing its crystallinity, whereas thermogravimetric analysis revealed a moderate decrease in degradation onset temperature that remained well above the processing and printing temperatures, ensuring safe fabrication. Tensile testing indicated that SCG incorporation led to noticeable reductions in filament strength and stiffness, whereas the elongation at break was only weakly affected because of counteracting plasticization effects. For the printed parts, SCGs imparted a dark brown coloration, decreased density, and increased moisture uptake due to their porous and hydrophilic nature, while tensile, flexural, and impact strengths were reduced and the tensile modulus and elongation at break remained statistically similar across the 0–20 wt% range. These findings indicate that SCGs can be effectively incorporated to tailor the crystallinity, color, and density of PLA-based 3D-printed composites, albeit with trade-offs in strength and impact performance.

## 1. Introduction

Material extrusion (MEX), also referred to as fused filament fabrication (FFF), is among the most widely employed additive manufacturing (AM) techniques and facilitates the layer-by-layer fabrication of parts directly from computer-aided design (CAD) models [1,2]. Because of its advantages of reduced material waste, high customizability, and lower time and cost compared with those of conventional plastic molding, FFF has been extensively applied in the automotive, manufacturing, food, biomedical, and aerospace sectors [2]. In daily life, the primary plastics commonly employed for such products are petrochemical-based polymers, such as polypropylene (PP), polyethylene (PE), polystyrene (PS), and polyamides (PAs), which are associated with severe environmental pollution [3]. Consequently, there is increasing interest in replacing these polymers with biodegradable materials for use in FFF-type 3D printers, including polycaprolactone (PCL), polybutylene succinate (PBS), and polylactic acid (PLA). The adoption of these biopolymers can reduce the dependence on fossil-based plastics, mitigate environmental impacts, and alleviate plastic pollution. Among them, PLA is a biomass-derived plastic produced from renewable resources such as corn, sugarcane, or wheat and offers several advantages, including environmental friendliness, biodegradability, and favorable biocompatibility, making it one of the most widely adopted feedstocks for 3D-printing filaments in recent years [4]. However, PLA also exhibits several drawbacks, such as relatively low thermal stability, susceptibility to hydrolysis, and intrinsic brittleness. To overcome these limitations, natural fibers with excellent mechanical properties, low density, suitable thermal stability, biocompatibility, biodegradability, and renewability, such as bamboo, wood, coconut, banana, and jute fibers, have been increasingly applied as reinforcements in PLA-based composites [5].

Coffee is among the most popular beverages worldwide, with a global production of 168.5 million 60 kg bags during the 2021–2022 crop year [6]. In addition to being rich in antioxidants, coffee contains various bioactive metabolites, including carotenoids, chlorogenic acids, phenolics, terpenes, and flavonoids, which contribute to a reduced risk of neurodegenerative diseases and lower oxidative stress [7]. Nevertheless, coffee production and consumption generate large quantities of byproducts, such as coffee silverskin and spent coffee grounds (SCGs). In recent years, numerous studies have focused on exploring valorization methods for these residues in packaging materials to reduce production costs and the environmental burden associated with landfilling coffee waste [8]. SCGs have also been used in vermicomposting, biorefinery processes, the extraction of valuable compounds, and the development of biocomposites [7,9,10,11,12,13]. Recently, the incorporation of SCGs into polymer matrices for FFF 3D printing has been investigated [14,15,16,17,18]. For example, Chang et al. (2019) [14] reported that the use of oil-extracted SCGs (20 wt%) greatly increased the impact toughness of printed PLA parts (25.24 MJ/m^3^) with only a modest decrease (26%) in the storage modulus, providing a low-cost, waste-derived 3D-printed filler. Moreover, Yu et al. (2023) [15] reported that food-derived byproducts such as SCGs and tea leaves can serve as sustainable fillers for PLA-based 3D-printed composites, thereby maintaining printability at a biomass content of 40 wt% while reducing the strength but greatly increasing ductility via natural oil plasticization and processing-dependent porosity control. As reported in a previous study [16], 3D-printed PLA parts containing SCGs maintained PLA-like thermal transitions while revealing optimal flexural strength and slightly increased tensile strength at an SCG content of 3 wt%, but the tensile strength notably decreased at SCG contents greater than 5 wt% because of filler-induced structural weakening. Lage-Rivera et al. (2024) [17] investigated the incorporation of lignin and SCGs into PLA filaments to increase sustainability, adjust rheological, thermal, mechanical, and water-uptake properties, and reduce the nozzle temperature. A composite part with a 15 wt% SCG content demonstrated notably increased elongation at break, a reduced printing temperature, and much higher water absorption, enabling moisture-intensive applications such as hydroponic systems; moreover, SCG application at low contents enhanced the degree of thermos-oxidative resistance, and lignin incorporation further enhanced thermal stability. Siala et al. (2025) [18] developed biodegradable, scented PLA biocomposite filaments for FFF by incorporating SCGs and lignin, as well as essential oils or microencapsulated fragrance compounds. Their results revealed tunable thermal, mechanical, hygroscopic, and sensory properties that are suitable for sustainable, multi-sensory design applications in 3D printing. However, although several studies have explored the incorporation of SCGs into PLA for FFF applications, most of these works have focused on specific aspects, such as mechanical performance, thermal behavior, or printability, without providing a comprehensive and systematic evaluation of both filament properties and the corresponding 3D-printed parts. In addition, the effects of SCG content on the process–structure–property relationships of PLA-based composites were insufficiently investigated, particularly in terms of the combined influence on filament fabrication, printability, and end-use performance. Within this context, the use of SCGs as filler material in FFF represents a promising strategy to combine waste valorization with the development of sustainable 3D-printed materials and to facilitate processability and commercialization through CAD-driven design. Accordingly, the objective of this study was to apply SCGs as a filler material in PLA to fabricate SCG–PLA composite (SPC) filaments and corresponding 3D-printed parts via FFF and to elucidate the influence of the SCG content on the physical and mechanical properties of both the filaments and 3D-printed SPC parts.

## 2. Experimental Section

### 2.1. Materials

In this study, PLA was employed as the polymer matrix, which was purchased from Magical Film Enterprise Co., Ltd. (Taichung, Taiwan) [19], with a melting temperature of 155 °C according to the supplier’s datasheet. SCGs were collected from a convenience store, dried in a hot-air oven at 105 °C for 72 h to remove moisture and achieve a constant mass, and subsequently milled using a grinder. The resulting material was sieved through a 100-mesh screen, corresponding to a nominal particle size below 150 μm to obtain SCG particles.

### 2.2. Preparation of SPC Filaments and Parts

**Figure 1:**
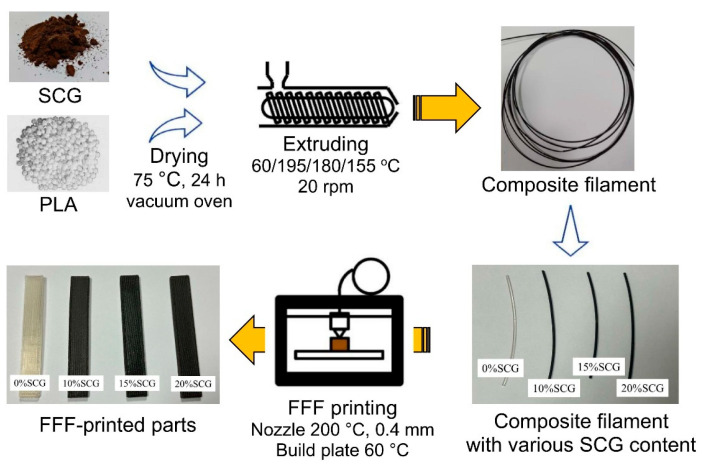
Scheme of manufacturing process of SPC filaments and 3D-printed parts.

**Figure 2:**
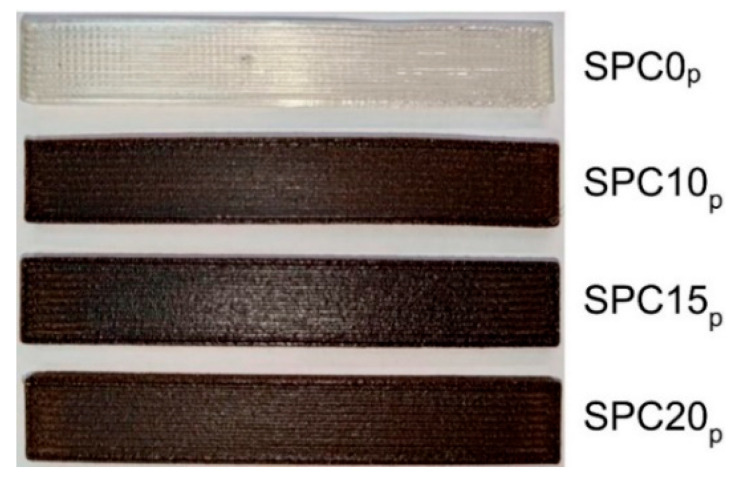
Appearances of 3D-printed SPC parts.

Before filament preparation was conducted, the experimental SCGs and PLA were dried in a vacuum oven at 75 °C for 24 h and subsequently melt-blended using a single-screw filament extruder (EX6 Filament Extruder, Filabot Co., Ltd., Barre, VT, USA). The extruder temperature range was divided into four zones, set from the feed zone to the melting/pumping zone at 60, 195, 180, and 155 °C. The extruder provided a screw speed of 20 rpm, and the extruded SPC filaments were cooled by a fan. To ensure material homogeneity, the compounding process was implemented twice, yielding filaments with a diameter of 1.65 ± 0.1 mm (Figure 1). Filaments with different SCG contents were produced by adjusting the SCG content to 0, 10, 15, and 20 wt%, designated as SPC0_f_, SPC10_f_, SPC15_f_, and SPC20_f_, respectively (Figure 1 and Figure 2). In addition, 3D-printed samples were fabricated with an FFF 3D printer (Creator Pro, Flashforge 3D Technology Co., Ltd., Jinhua, China). The printing parameters were as follows: a nozzle diameter of 0.4 mm, a nozzle temperature of 200 °C, a build-plate temperature of 60 °C, a printing speed of 20 mm/s, and a layer height of 0.2 mm. On the basis of the SCG content, the printed parts were denoted SPC0_p_, SPC10_p_, SPC15_p_, and SPC20_p_. Before property testing was conducted, all the printed parts were conditioned at a temperature of 20 °C and a relative humidity of 65% for 12 days.

### 2.3. Characterization

#### 2.3.1. Density

In accordance with ASTM D792 [20], the density (*ρ*) of the printed SPC parts (dimensions: 20 mm × 10 mm × 5 mm) was determined via the Archimedes water displacement method using a semimicro analytical balance (GH-200, A&D Co., Ltd., Tokyo, Japan). Prior to testing, the masses of all the samples (m_A_) were measured after conditioning at a temperature of 20 °C and a relative humidity of 65% for 12 days. The samples were then immersed in water at 23 °C, and their mass in water (m_w_) was recorded. The density was calculated via the following equation, and five replicates were analyzed for each formulation.

*ρ* (kg/m^3^) = (m_A_ × δ_w_)/(m_A_ − m_w_) (1)

where m_w_ is the mass after immersion in water (g), m_A_ is the mass before immersion in water (g), and δ_w_ is the density of water at 23 °C.

#### 2.3.2. Surface Color

The color parameters of the SPC parts were measured with an ultraviolet (UV)–visible (Vis)–near-infrared (NIR) spectrophotometer (LAMBDA 1050+, PerkinElmer Co., Ltd., Waltham, MA, USA). The color difference (ΔE*) was calculated as follows [21]:

ΔE* = [(L*_1_ − L*_0_)^2^ + (a*_1_ − a*_0_)^2^ + (b*_1_ − b*_0_)^2^ ]^1/2^ (2)

where L*_1_ and L*_0_ are the lightness values of the SPC parts without and with SCGs, respectively, a*_1_ and a*_0_ are the red/green coordinates of the SPC parts without and with SCGs, respectively, and b*_1_ and b*_0_ are the yellow/blue coordinates of the SPC parts without and with SCGs, respectively.

#### 2.3.3. Tensile Properties

**Figure 3:**
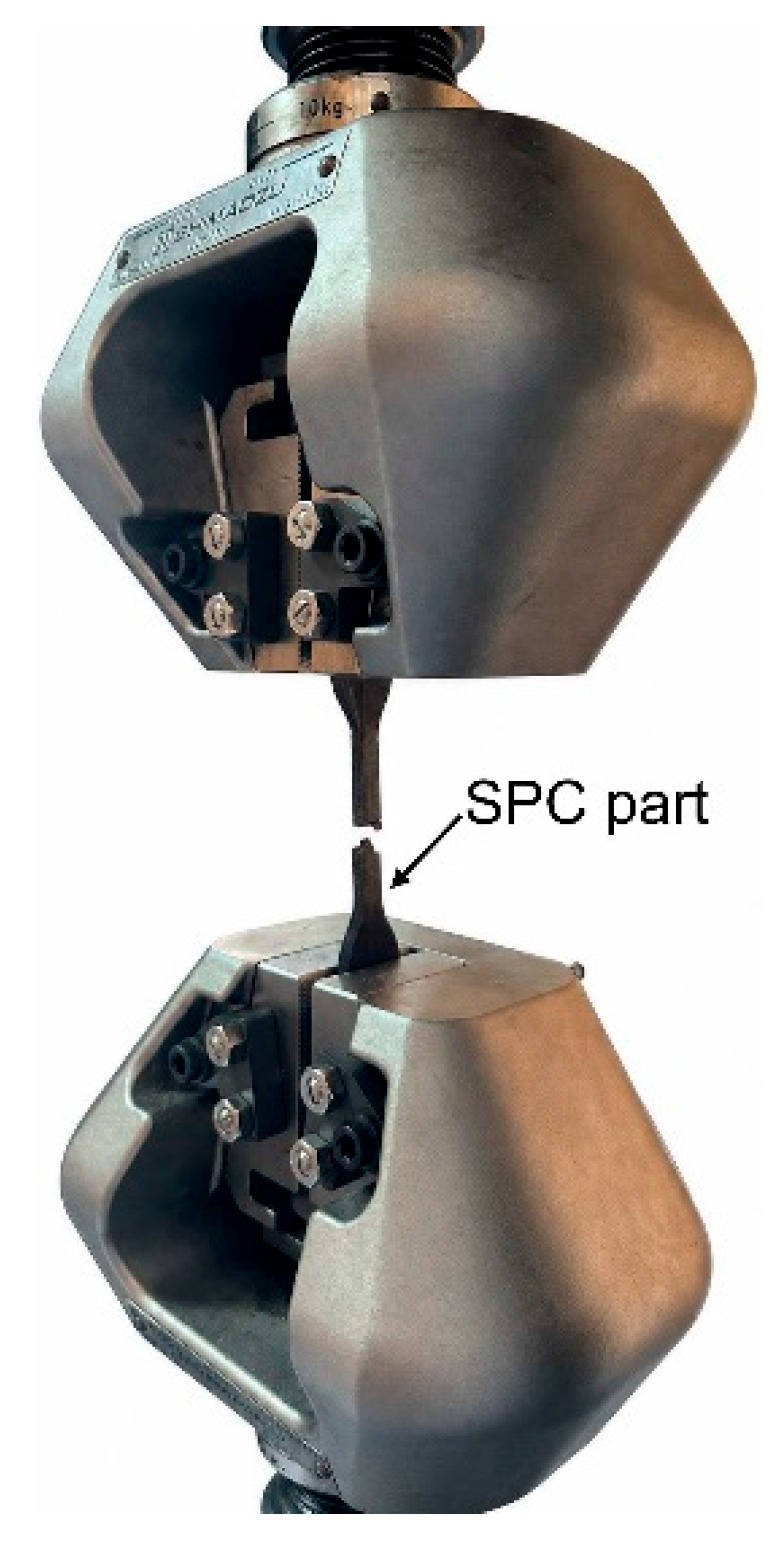
Fixtures of the tensile test and setup of the SPC part.

Tensile test (Figure 3) was conducted in accordance with ASTM D638 [22]. The SPC parts were 3D-printed into a Type-IV geometry and analyzed at a crosshead speed of 5 mm/min with an initial gauge length of 65 mm to determine the tensile strength (*σ*_tp_), tensile modulus (*E*_tp_), and elongation at break (*ε*_tp_). Three replicates were analyzed for each formulation. Tensile tests were also conducted for the filaments, with an initial gauge length of 30 mm and a crosshead speed of 450 mm/min, to obtain the tensile strength (*σ*_tf_), tensile modulus (*E*_tf_), and elongation at break of the filaments (*ε*_tf_). In this case, six samples were analyzed for each condition. The above tensile properties were calculated as follows:

*σ*_t_ (MPa) = *F_u_*/*A* (3)

*E*_t_ (GPa) = Δ*σ*_t_/Δ*ε*_t_ (4)

*ε*_t_ (%) = *δ*/*L* × 100 (5)

where *F_u_* is the ultimate load (N), *A* is the cross-sectional area (mm^2^), *δ* is the elongation at break (mm), *L* is the initial gauge length (mm), Δ*σ*_t_ is the difference between the upper and lower stress values within the proportional limit (MPa), and Δ*ε*_t_ is the corresponding strain difference.

#### 2.3.4. Flexural Properties

**Figure 4:**
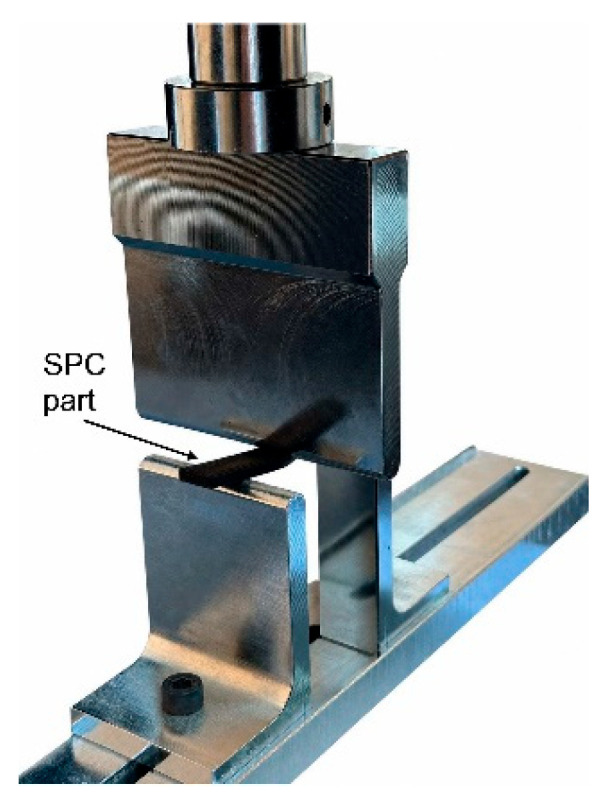
Fixtures of the flexural test and setup of the SPC part.

Flexural test (Figure 4) was conducted in accordance with ASTM D790 [23] using a three-point flexural test method. The span length was set to 16 times the sample thickness, and the crosshead speed was 1.28 mm/min. The ultimate load and mid-span deflection were recorded and employed to calculate the modulus of rupture (MOR) and the modulus of elasticity (MOE). The sample dimensions were 80 mm × 12.7 mm × 3.2 mm, and three samples were analyzed for each condition.

MOR (MPa) = 3*F_u_L*/(2*bh*^2^) (6)

MOE (GPa) = 3Δ*FL*^3^/(4Δ*Ybh*^3^) (7)

where *F_u_* is the ultimate load (N), *L* is the support span (mm), *b* is the sample width (mm), *h* is the sample thickness (mm), Δ*F* is the difference between the upper and lower loads within the proportional limit (N), and Δ*Y* is the corresponding mid-span deflection (mm).

#### 2.3.5. Impact Strength

**Figure 5:**
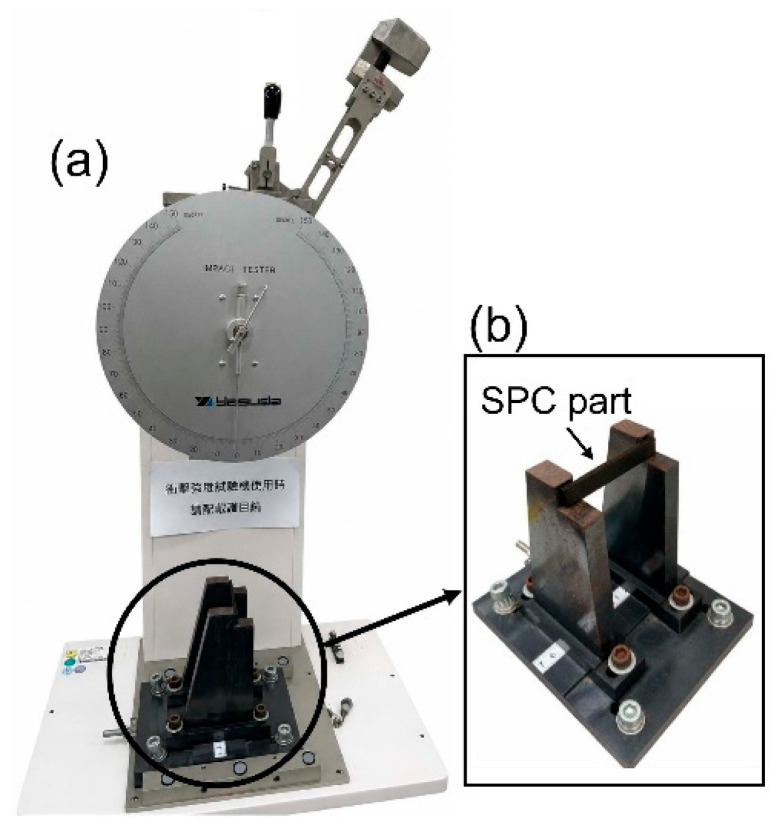
Appearance of the impact tester (**a**) and setup of the SPC part (**b**).

The Charpy impact strength (IS) of the SPC parts was evaluated in accordance with CNS 5846-1 [24] on the basis of unnotched rectangular samples with dimensions of 80 mm × 10 mm × 4 mm, which were analyzed on a YASUDA impact tester (Figure 5). The IS value was calculated as follows:

IS (kJ/m^2^) = *E*_c_/(*bh*) × 10^3^ (8)

where *E*_c_ is the absorbed energy (J), *h* is the sample thickness (mm), and *b* is the sample width (mm).

#### 2.3.6. Differential Scanning Calorimetry (DSC)

The thermal behavior of the SPC filaments (approximately 5 mg) was analyzed with a differential scanning calorimeter (DSC 8500, PerkinElmer, Beaconsfield, UK) to determine the glass transition temperature (T_g_), crystallization temperature (T_c_), and melting temperature (T_m_). The samples were heated from 20 to 210 °C at a rate of 10 °C/min under a nitrogen atmosphere with a flow rate of 20 mL/min. The crystallinity index (*X*_c_) of the PLA matrix was calculated as follows [25]:

*X*_c_ (%) = (Δ*H*_m_ − Δ*H*_cc_)/(Δ*H*^0^ × *w*_c_) (9)

where Δ*H*_m_ is the melting enthalpy, Δ*H*_cc_ is the enthalpy of cold crystallization, Δ*H*^0^ is the melting enthalpy of 100% crystalline PLA (93 J/g), and *w*_c_ is the weight fraction of the PLA matrix.

#### 2.3.7. Thermogravimetric Analysis (TGA)

Thermogravimetric analysis (TGA) of the SPC filaments was conducted in accordance with ASTM E1131 via the use of a thermogravimetric analyzer (Pyris 1 TGA, PerkinElmer Co., Ltd., USA). Filament samples (10 ± 0.5 mg) were heated from 50 to 600 °C at a heating rate of 10 °C/min under a nitrogen atmosphere with a flow rate of 20 mL/min to evaluate their thermal stability.

#### 2.3.8. Scanning Electron Microscopy (SEM)

The failure cross-sectional surfaces of the SPC filaments and printed samples after tensile testing were examined under a scanning electron microscope (TM-1000, Hitachi, Tokyo, Japan) operated at an accelerating voltage of 15 kV.

### 2.4. Analysis of Variance

The statistical significance of the differences among all SPC samples was evaluated using Scheffé’s test at a significance level of *p* < 0.05 [26].

## 3. Results and Discussion

### 3.1. Properties of SPC Filaments

#### 3.1.1. Thermal Properties

**Figure 6:**
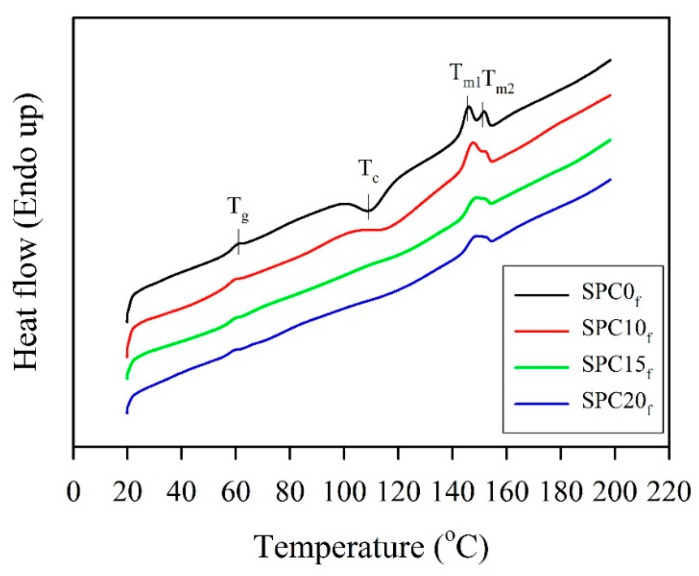
DSC curves of the SPC filaments with various SCG contents.

DSC curves were obtained in the heating process of the SPC filaments. Notable, the glass transition temperature (T_g_), cold crystallization temperature (T_c_), and melting temperature (T_m_) were determined, and the results are shown in Figure 6. As summarized in Table 1, the T_g_ value of the neat PLA filament (SPC0_f_) was 60.5 °C, whereas that of all the SPC filaments containing SCGs exhibited slightly lower T_g_ values (approximately 59.7 °C). This marginal decrease suggests that SCG incorporation had a limited plasticizing effect; however, the differences were not significant among the composite filaments with SCGs. During continuous heating, SPC0_f_ exhibited an exothermic peak (T_c_ = 109.9 °C) associated with cold crystallization, resulting from the increased mobility and subsequent rearrangement of PLA macromolecular chains. At an SCG content of 10 wt% (SPC10_f_), the T_c_ value of the composite filament was higher (115.5 °C) than that of SPC0_f_, indicating that SCG incorporation notably impeded the cold crystallization of PLA. When the SCG content exceeded 10 wt%, no distinct T_c_ value could be observed for SPC15_f_ and SPC20_f_. In addition, the neat PLA filament exhibited two distinct melting temperatures (T_m1_ = 145.9 °C and T_m2_ = 151.9 °C), where the former (T_m1_) corresponds to the melting of the original crystals in the filament and the latter (T_m2_) can be attributed to crystals formed via melt–recrystallization during heating [12,27]. When 10 wt% SCGs was incorporated, the melting temperatures T_m1_ and T_m2_ increased to 147.0 and 154.3 °C, respectively. Furthermore, with a continued increase in the SCG content above 10 wt% (SPC15_f_ and SPC20_f_), the T_m1_ value increased slightly, whereas the T_m2_ value disappeared. This phenomenon may be attributed to the nucleating effect of SCGs on PLA crystallization during filament extrusion, leading to the formation of more stable crystals to suppress recrystallization during DSC heating scan.

As further shown in Table 1, the degree of crystallinity (X_c_) of PLA increased notably upon SCG incorporation. The X_c_ value increased from 6.8% for neat PLA to over 20% for all SCG-containing filaments. The increase in the X_c_ value at higher SCG contents can be attributed to the combined effects of heterogeneous nucleation provided by SCG particles and the presence of low-molecular-weight extractives. SCGs provide abundant nucleation sites, thereby promoting the formation of PLA crystals, while the extractable components may partially migrate into the PLA matrix during extrusion, locally increasing chain mobility and further facilitating crystallization [12]. With increasing SCG content, the combined contribution of particle-induced nucleation and extractive-mediated enhancement led to a substantial increase in crystallinity up to 10 wt%. Beyond an SCG content of 10 wt%, the degree of crystallinity approached a quasiplateau (approximately 21–23%) because of the increasing restrictions on chain mobility and crystal growth imposed by the higher SCG content. This interpretation indicates that both the particulate phase and SCG extractives synergistically govern the crystallization behavior of PLA in the SPC filaments.

**Figure 7:**
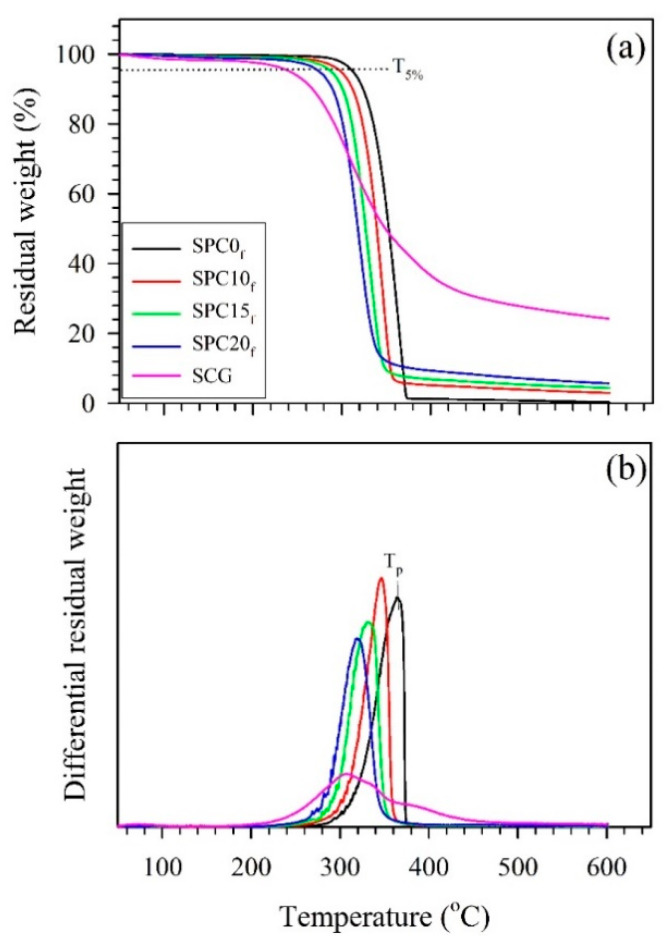
TGA curves of the SPC filaments with various SCG contents: (**a**) Residual weight and (**b**) Differential residual weight.

The TGA curves of the SPC filaments are shown in Figure 7, and various TGA parameters are listed in Table 2. Compared with that of neat PLA (SPC0_f_), the temperature at a 5 wt% residual weight (T_5%_) of the filaments clearly decreased, and the T_5%_ value decreased with increasing SCG content (Table 2). This phenomenon conforms to the obtained peaks for different residual weights (T_p_) shown in Figure 7b. These findings revealed that the degree of thermal stability decreased upon SCG addition. This reduction was related to several concurrent factors associated with the incorporation of SCGs into the PLA matrix. First, SCGs comprise mainly lignocellulosic components with residual oils and other organic substances, all of which typically exhibit onset degradation temperatures lower than that of neat PLA (Figure 7b). Consequently, the early thermal degradation of SCGs during heating causes an apparent decrease in the onset decomposition temperature of the composites, rendering the overall SPCs less thermally stable than neat PLA [14,17]. In addition, the intrinsic hydrophilicity and porous structure of SCGs promote moisture uptake, and residual water may remain in the SCGs even after drying. In the TGA tests, this residual moisture can induce hydrolytic degradation of PLA to contribute to the earlier onset of mass loss [27]. Moreover, as described in the DSC results, the addition of SCGs can enhance the crystallinity of PLA through a nucleating effect, which would normally be expected to enhance thermal resistance. However, this beneficial contribution may be outweighed by the lower thermal stability of the lignocellulosic filler material and moisture-induced hydrolysis, resulting in a reduced thermal stability in the TGA curve compared with that of SPC0_f_. This drawback is not expected to affect material performance during filament extrusion or 3D printing because the processing temperatures employed for both operations remain below 200 °C (Figure 7b).

#### 3.1.2. Tensile Properties

**Figure 8:**
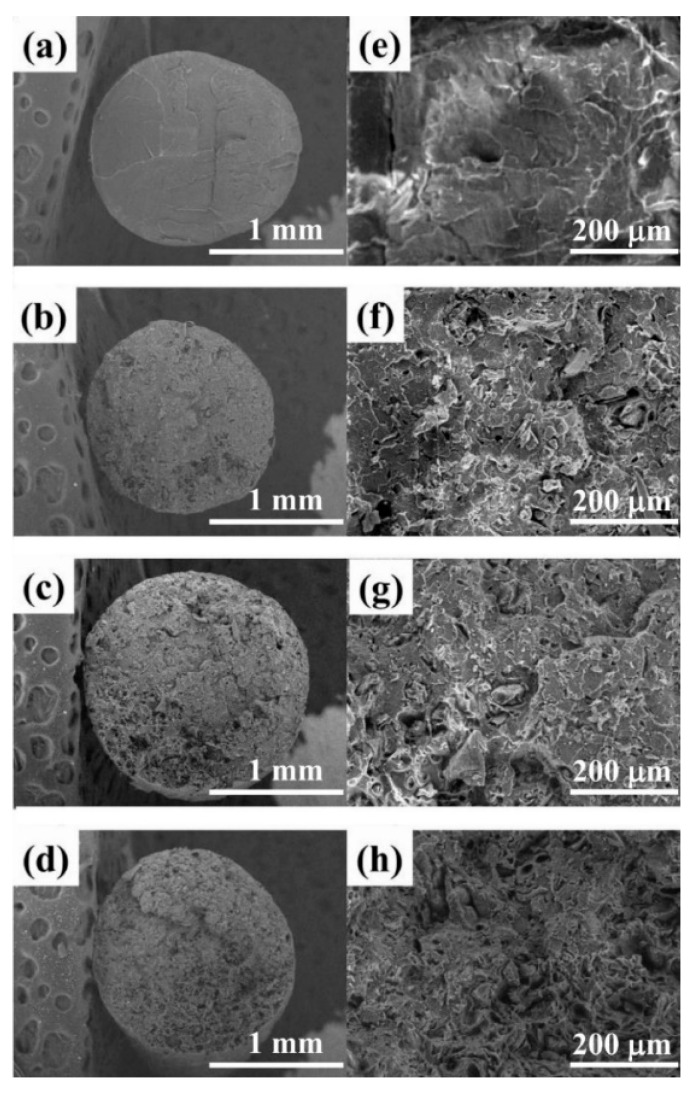
Failure cross section of the SPC filaments with various SCG contents after tensile test: (**a**,**e**) SPC0_f_, (**b**,**f**) SPC10_f_, (**c**,**g**) SPC15_f_, and (**d**,**h**) SPC20_f_. (**a**–**d**) low magnification and (**e**–**h**) high magnification of SEM images.

The tensile properties of the SPC filaments with various SCG contents are summarized in Table 3. With respect to the neat PLA filament without SCGs (SPC0_f_), the tensile strength (*σ*_tf_) and tensile modulus (*E*_tf_) were 70.1 MPa and 1.4 GPa, respectively. As the SCG content was increased to 20 wt%, compared with those of SPC0_f_, the *σ*_tf_ and *E*_tf_ values significantly decreased by 41.8% and 28.6%, respectively. However, the elongation at break of the filaments (*ε*_tf_) was not significantly affected by SCG addition, and the values ranged from 7.2 to 8.3%. The influences of SCG loading on the fracture morphology and internal structure of the filaments were examined, and fracture cross sections of the SPC filaments with different SCG contents after tensile testing are shown in Figure 8. As shown in Figure 8a,e, the fracture surface of the neat PLA filament was relatively smooth. In contrast, upon the incorporation of SCGs into the PLA matrix, the emergence of pores caused by SCG pull-out and a rougher fracture surface could be clearly observed. As shown in Figure 8d,h, when the SCG content was increased to 20 wt%, more notable SCG agglomeration and a significant increase in pores were observed. This phenomenon suggests low interfacial compatibility between the SCGs and the PLA matrix, resulting in weak interfacial interactions between the two phases [12]. These results indicated that the *σ*_tf_ and *E*_tf_ values of the SPC filaments decreased with increasing SCG content, mainly because of SCG agglomeration and low interfacial adhesion between the SCGs and the PLA matrix [9]. In general, the incorporation of natural fillers into PLA reduces the elongation at break of the resulting composites [28]; however, in this study, SCG addition did not adversely affect the elongation at break, regardless of the SCG content. This behavior can be attributed to the presence of oily and other extractable substances in the SCGs, which can serve as plasticizing components and thereby enhance the deformability of the PLA matrix [15,17]. As a result, the presence of SCGs counterbalances, and may even overcome, the typical decrease in elongation at break attributed to the introduction of rigid fillers, yielding comparable or slightly increased ductility of the composites.

### 3.2. Properties of the Printed SPC Parts

#### 3.2.1. Surface Color

The color parameters of the printed SPC parts with various SCG contents are provided in Table 4. The lightness coordinate (L*) of the printed SPC parts significantly decreased from 43.8 to 25.6 with increasing SCG content. Additionally, the red/green coordinates (a* = 2.7) and blue/yellow coordinates (b* = 3.3) were highest for the printed SPC parts with a 15 wt% SCG content, whereas the a* and b* values decreased to 2.0 and 1.6, respectively, at a SCG content of 20 wt%. The decrease in all color parameters for the SPC parts with SCG contents of 10–15 wt% resulted in a notable dark brown color, which can be primarily attributed to organic compounds generated by Maillard reactions during coffee bean roasting [13,29,30]. When the SCG content was further increased to 20 wt%, the decrease in all color parameters indicated a shift from a deep brown tone to a duller, brownish-gray hue. This reduction in chromatic coordinates at higher SCG contents can be attributed to the increased proportion and agglomeration of dark SCG particles, which enhances light scattering and reduces color saturation. Furthermore, compared with neat PLA (SPC0_p_), the SPC parts exhibited very large color difference values (ΔE* values ranging from 76.7 to 84.0) regardless of the SCG content, indicating a notable color variation that is readily perceptible to the naked eye.

#### 3.2.2. Density and Hygroscopicity

**Figure 9:**
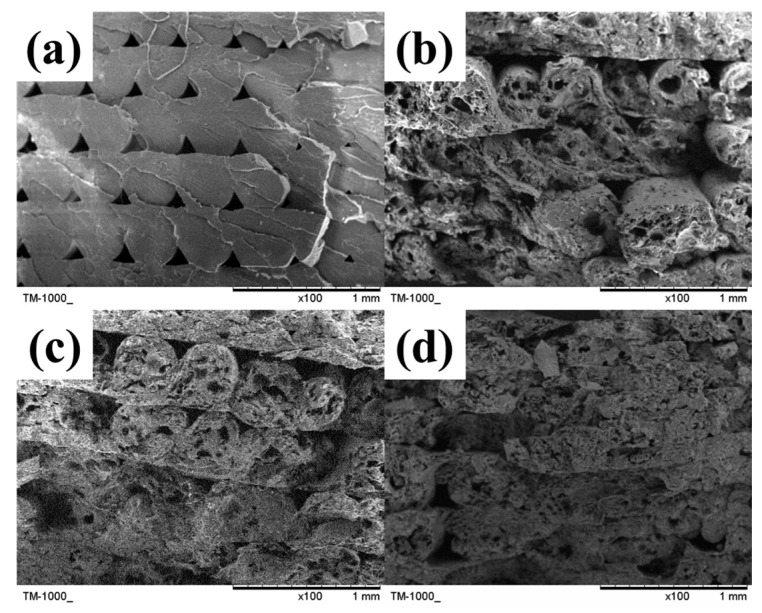
Failure cross section of the printed SPC parts with various SCG contents after tensile test: (**a**) SPC0_p_, (**b**) SPC10_p_, (**c**) SPC15_p_, and (**d**) SPC20_p_.

As indicated in Table 5, the density (*ρ*) of SPC0_p_ was 1127 kg/m^3^. Upon the addition of SCGs, the *ρ* values of all the printed SPC parts containing SCGs were lower than that of SPC0_p_, but there were no significant differences among all the SPC parts within the range of 979–1072 kg/m^3^. SEM images of the fracture cross sections of the SPC parts after tensile testing are shown in Figure 9. The SPC part without SCGs exhibited a relatively smooth cross-sectional surface (Figure 9a), which can be attributed to the inherently brittle nature of PLA. In addition, distinct pores between the extruded filaments originating from the printing nozzle were clearly observed. These pores are closely related to the nozzle diameter and the selected layer height in the FFF process. Furthermore, several factors can account for the relatively low density of the SPC parts containing SCGs (Figure 9b–d): (1) SCG exhibits low density, with a reported value of approximately 450 kg/m^3^ [31]; (2) pores are present in the extruded filaments; and (3) pores exist between adjacent extruded filaments. Despite the overall lower density of the SPC parts containing SCGs, an interesting observation is that there was no significant difference in density among the SPC parts containing 10–20 wt% SCGs. This behavior may result from the compensation between two opposing effects. With increasing SCG content, the density of the SPC part decreases because of the intrinsically low density of SCGs [12,16]. In contrast, the addition of SCGs, which contain coffee oil, can increase the melt flowability of the filament during printing [13], thereby reducing the number of pores in and between the extruded filaments; this effect typically increases the density of the printed parts at higher SCG contents.

The hygroscopic behavior of the SPC parts, in terms of the moisture content (MC), was governed by a combination of their morphological characteristics and the intrinsic properties of the polymer matrix and lignocellulosic fillers employed [32]. The MC values of the printed SPC parts with various SCG contents are provided in Table 5. PLA is inherently hydrophilic. However, the SPC part without SCGs (SPC0_p_) attained the lowest MC value (0.5 wt%) among all the samples. When the SCG content was increased from 10 to 20 wt%, the MC values of the SPC parts significantly increased from 0.79% to 1.36%. SCGs possess a highly porous microstructure with numerous microscopic pores, which promotes the penetration and retention of water within the filler phase. In addition, the hygroscopic constituents of SCGs contain abundant hydroxyl groups that can form hydrogen bonds with water molecules, thereby increasing the overall moisture capacity of the resulting SPC parts [9,17,18]. In addition, the pores formed between adjacent extruded filaments within the SPC parts significantly contribute to an increase in water absorption. However, in this study, the printing strategy involved the deposition of a continuous outer contour, which effectively shielded the internal structure from direct exposure, resulting in no significant increase in MC.

#### 3.2.3. Mechanical Properties and Impact Strength

The tensile and flexural properties of the printed SPC parts with various SCG contents are summarized in Table 5. Compared with the tensile properties of SPC0_p_, the tensile strength (*σ*_tp_) of the SPC parts significantly decreased from 50.6 to 16.7 MPa with increasing SCG content, whereas the tensile modulus (*E*_tp_) and elongation at break (*ε*_tp_) of the SPC parts ranged from 1.9 to 2.8 GPa and from 2.7 to 4.2%, respectively. According to previous studies [15,16,18,33], the decrease in the tensile strength of composite parts can be attributed to the presence of ester groups in the coffee oil contained within the SCGs, which not only promotes the agglomeration of SCG particles but also attenuates the interfacial bonding between the matrix and the filler. As a result, the tensile strength of the printed parts decreases significantly with increasing SCG content. With respect to printed parts containing up to 20 wt% SCGs, the absence of statistically significant differences in the tensile modulus and elongation at break can be explained by the superposition of several competing effects. First, the coffee oil present in SCGs, together with moisture within the printed parts, can function as a plasticizing agent, which can decrease the tensile modulus and increase the elongation at break by enhancing the chain mobility and deformability of the PLA matrix [15,18,34]. In contrast, SCG incorporation promotes an increase in the crystallinity of the PLA matrix (Table 1), which typically results in a higher tensile modulus and a lower elongation at break [35,36]. As these antagonistic contributions effectively counterbalance each other across the investigated SCG loading range, the net result is that the tensile modulus and elongation at break of the SPC parts do not significantly vary with increasing SCG content.

As indicated in Table 5, the MOR and MOE values of SPC0_p_ were 98.9 MPa and 2.7 GPa, respectively. Compared with that of SPC0_p_, the MOR value of the SPC part containing 20 wt% SCGs significantly decreased by 47.4%. The MOE values of the SPC parts exhibited a nonmonotonic dependence on the SCG content. Specifically, the MOE value initially decreased to 2.1 GPa at a 10 wt% SCG content, then increased to 2.8 GPa at a 15 wt% SCG content, and finally decreased to 1.8 GPa at a 20 wt% SCG content. The flexural response reflects the combined contribution of the tensile and compressive zones under bending. As described above, the tensile modulus of the SPC parts did not significantly vary with SCG content. Therefore, the observed variations in the MOE values are likely governed primarily by changes in the compression zone. The combined effect of the intrinsically high rigidity of SCG particles and the increased crystallinity of the PLA matrix increase the compressive modulus in the compression zone, resulting in an increase in the MOE value of the SPC parts. Furthermore, at high SCG contents (SPC20_p_), the reduction in the MOE value can be attributed mainly to the high content of coffee oil components, which increases the flexibility of the material [18]. With respect to the impact resistance (Table 5), the IS values of the SPC parts significantly decreased upon the incorporation of SCGs. The addition of 10 wt% SCG decreased the IS value of the parts, with values increasing from 33.7 kJ/mm^2^ for the parts without SCGs (SPC0_p_) to 20.2 kJ/mm^2^. As the SCG content was increased to 20 wt%, the IS value further decreased by 65.6%. This deterioration can be attributed primarily to the limited interfacial adhesion between the SCG fillers and the PLA matrix, which promotes filler debonding, pore formation, and microcrack initiation under dynamic loading [12]. The presence of structural defects and agglomerated filler domains at increased filler concentrations facilitates crack propagation, thereby compromising the ability of the material to dissipate impact energy [32,33].

## 4. Conclusions

The main conclusions of this study are summarized as follows:

- SCGs were incorporated into PLA-based filaments to print SPC parts via FFF, and their structure–property relationships were systematically clarified.
- The DSC results demonstrated that SCGs functioned as an effective heterogeneous nucleating agent, thereby increasing the crystallinity of PLA from a low level in the neat filament to greater than 20% at SCG contents of 10–20 wt%.
- The TGA results revealed that the overall thermal stability of the SPC filaments decreased with increasing SCG content, mainly because of the earlier degradation of the lignocellulosic SCG phase and moisture-induced hydrolysis, although this reduction remains acceptable for typical filament extrusion and FFF processing temperatures.
- SCG addition caused a clear decrease in the tensile strength and modulus for both the filaments and printed parts, as well as a significant deterioration in the IS and flexural strength at high SCG loadings. These effects could be attributed to SCG agglomeration, low interfacial adhesion, and defect formation.
- The elongation at break of the filaments and printed parts did not significantly decrease, suggesting that coffee oil and other extractives in SCGs provide a plasticizing effect that counterbalances the embrittlement normally caused by the incorporation of rigid fillers and increased crystallinity.
- The incorporated SCGs resulted in a distinctive dark brown coloration, reduced density, and a moderate increase in the hygroscopicity of the printed parts.
- The combination of tailored esthetics, partial weight reduction, and the valorization of a ubiquitous biomass waste highlights the potential of SPCs for decorative or nonstructural components produced by FFF.

Overall, SCGs constitute a promising sustainable filler material for PLA-based filaments, with the provision of tunable crystallinity, appearance, and density, but its adverse effects on strength and impact performance must be considered in the design of end-use applications or mitigated through interfacial modification and optimized processing.

## Figures and Tables

**Table 1:** DSC results and crystallinity of the SPC filaments with various SCG contents.

Code	SCG Content (wt%)	T_g_ (°C)	T_c_ (°C)	T_m1_ (°C)	T_m2_ (°C)	X_c_ (%)
SPC0_f_	0	60.5	109.9	145.9	151.9	6.8
SPC10_f_	10	59.7	115.5	147.0	154.3	22.1
SPC15_f_	15	59.7	-	148.3	-	21.7
SPC20_f_	20	59.7	-	148.3	-	23.4

-: No data detected.

**Table 2:** TGA results of the SPC filaments with various SCG contents.

Code	SCG Content (%)	T_5%_ (°C)	T_p_ (°C)
SCG		242.0	307.1
SPC0_f_	0	312.6	363.6
SPC10_f_	10	299.2	341.8
SPC15_f_	15	288.5	330.5
SPC20_f_	20	274.6	319.2

**Table 3:** Tensile properties of the SPC filaments with various SCG contents.

Code	SCG Content (%)	*σ*_tf_ (MPa)	*E*_tf_ (GPa)	*ε*_tf_ (%)
SPC0_f_	0	70.1 ± 2.9 ^a^	1.4 ± 0.1 ^a^	8.3 ± 1.2 ^a^
SPC10_f_	10	57.3 ± 3.9 ^b^	1.2 ± 0.1 ^a^	7.3 ± 0.6 ^a^
SPC15_f_	15	43.8 ± 3.0 ^c^	1.0 ± 0.2 ^b^	7.2 ± 0.8 ^a^
SPC20_f_	20	40.8 ± 1.5 ^c^	1.0 ± 0.1 ^b^	7.4 ± 0.5 ^a^

Values are the mean ± SD (*n* = 6). Different letters within a column indicate significant differences (*p* < 0.05).

**Table 4:** Color parameters of the printed SPC parts with various SCG contents.

Code	SCG Content (%)	L*	a*	b*	ΔE*
SPC0_p_	0	43.8 ± 1.3 ^a^	−0.5 ± 0.1 ^c^	1.8 ± 0.6 ^ab^	-
SPC10_p_	10	27.1 ± 0.5 ^b^	2.3 ± 0.1 ^ab^	2.6 ± 0.2 ^ab^	76.7 ± 2.6 ^a^
SPC15_p_	15	27.5 ± 0.7 ^b^	2.7 ± 0.4 ^a^	3.3 ± 0.9 ^a^	78.9 ± 3.1 ^a^
SPC20_p_	20	25.6 ± 0.3 ^b^	2.0 ± 0.1 ^b^	1.6 ± 0.2 ^b^	84.0 ± 2.4 ^a^

Values are the mean ± SD (*n* = 3). Different letters within a column indicate significant differences (*p* < 0.05).

**Table 5:** Density (*ρ*), moisture content (MC), tensile properties, flexural properties, and impact strength (IS) of the printed SPC parts with various SCG contents.

Code	SCG Content (%)	*ρ* (kg/m^3^)	MC (%)	Tensile Properties			Flexural Properties		IS (kJ/mm^2^)
				*σ*_tp_ (MPa)	*E*_tp_ (GPa)	*ε*_tp_ (%)	MOR (MPa)	MOE (GPa)	
SPC0_p_	0	1127 ± 31 ^a^	0.50 ± 0.18 ^c^	50.6 ± 2.0 ^a^	1.9 ± 0.8 ^a^	3.1 ± 0.1 ^a^	98.9 ± 3.4 ^a^	2.7 ± 0.2 ^a^	33.7 ± 1.4 ^a^
SPC10_p_	10	1019 ± 79 ^b^	0.79 ± 0.22 ^bc^	30.2 ± 7.9 ^b^	2.8 ± 0.2 ^a^	2.7 ± 0.7 ^a^	56.2 ± 1.9 ^bc^	2.1 ± 0.1 ^b^	20.2 ± 2.1 ^b^
SPC15_p_	15	979 ± 74 ^b^	1.13 ± 0.31 ^ab^	22.1 ± 3.9 ^bc^	2.1 ± 0.4 ^a^	4.2 ± 1.1 ^a^	60.8 ± 0.6 ^b^	2.8 ± 0.0 ^a^	12.9 ± 1.2 ^c^
SPC20_p_	20	1072 ± 25 ^ab^	1.36 ± 0.09 ^a^	16.7 ± 1.8 ^c^	2.0 ± 0.3 ^a^	3.0 ± 1.0 ^a^	52.0 ± 1.3 ^c^	1.8 ± 0.1 ^b^	11.6 ± 0.3 ^c^

Values are the mean ± SD (*n* = 5 for density, *n* = 3 for MC, tensile properties, flexural properties, and impact strength). Different letters within a column indicate significant differences (*p* < 0.05).

## Data Availability

The datasets presented in this article are not readily available because the data are part of an ongoing study. Requests to access the datasets should be directed to the corresponding author.
